# Estimating the Postmortem Interval of Carcasses in the Water Using the Carrion Insect, Brain Tissue RNA, Bacterial Biofilm, and Algae

**DOI:** 10.3389/fmicb.2021.774276

**Published:** 2022-01-04

**Authors:** Yu Wang, Man Wang, Wang Xu, Yinghui Wang, Yanan Zhang, Jiangfeng Wang

**Affiliations:** Department of Forensic Medicine, Soochow University, Suzhou, China

**Keywords:** forensic entomology, postmortem interval, RNA degradation, bacterial biofilm, algae

## Abstract

The accurate estimation of postmortem interval (PMI) is crucial in the investigation of homicide cases. Unlike carcasses on land, various biological and abiotic factors affect the decomposition of carcasses in water. In addition, the insect evidence (e.g., blow flies) that is commonly used to estimate the PMI are unavailable before the carcasses float on water. Therefore, it is difficult to estimate the PMI of a carcass in water. This study aimed to explore an effective way of estimating the PMI of a carcass in water. Carrion insects, brain tissue RNA, bacterial biofilm on the skin surface, and algae in water with PMI were studied using 45 rat carcasses in a small river. The results showed that carrion insects might not be suitable for the estimation of PMI of a carcass in water since they do not have a regular succession pattern as a carcass on land, and the flies only colonized six of the carcasses. The target genes (β-actin, GAPDH, and 18S) in the brain tissue were associated with the PMI in a time-dependent manner within 1 week after death. A polynomial regression analysis was used to assess the relationship between the gene expression profiles and PMI. The correlation coefficient *R*^2^ of each regression equation was ≥ 0.924. A third-generation sequencing analysis showed that the bacteria on the skin surface of the carcass and the algae in the water samples around the carcass had a regular succession pattern, where *Cryptomonas* and *Placoneis* incased and decreased, respectively, within first 9 days. The results of this study provide a promising way to use the brain tissue RNA, bacterial biofilm, and algae to estimate the PMI of a carcass in water.

## Introduction

Unlike carcasses on land, various biological and abiotic factors, such as temperature, water depth, and aquatic organisms, affect the decomposition of carcasses in water (Sorg and Haglund, 1996). There are almost no obligate carrion insects in water (Haskell et al., 1989). Thus aquatic insects usually cannot be used as the indicators to estimate the postmortem interval (PMI) of submerged carcasses. The body of water also blocks most terrestrial sarcosaprophagous insects, making it difficult for them to reach the carcass (Merritt and Wallace, 2009). Even if a few insects can colonize the carcass during the floating stage, the insect evidence can only provide the PMI after the carcass has started floating, which is usually different from the real PMI owing to the uncertainty of the time at which it is submerged (Amendt et al., 2007; Merritt and Wallace, 2009). Furthermore, the rolling and mobility of carcasses owing to the water flow could increase the complexity of insect evidence (Barrios and Wolff, 2011). All of the factors mentioned make it difficult to estimate the PMI of a carcass in water, and methods of reliably estimating PMI are urgently needed for submerged carcasses.

Previous studies have indicated that degradation of some RNA or the loss of transcription of specific RNA in cadaveric tissue can indicate PMI (Ren et al., 2009; Wu et al., 2010; Deng et al., 2013; Sampaio-Silva et al., 2013; Young et al., 2013; Elghamry et al., 2017). However, no study has reported on the changes in carcass RNA in water. Brain tissue is relatively less affected by the outside environment and is commonly used to analyze the degradation of RNA. Inoue et al. (2002) first explored the relationship between RNA degradation and PMI and found that various carcass tissues have different rates of degradation of RNA. Moreover, they showed that the RNA is most stable in brain tissues. To date, several researchers have assessed the pattern of degradation of RNA in brain tissue and analyzed the relationship between different marker genes and PMI (Liu et al., 2007; Ren et al., 2009; Zhu et al., 2011; Ma et al., 2015; Lv et al., 2016a,^2017^; Elghamry et al., 2017) and found that GAPDH, β-actin, and 18S genes are associated with PMI. Water has a high specific heat capacity and stable temperature. Therefore, the pattern of degradation of RNA could be more suitable to determine the PMI in water carcasses than those on land.

Bacteria in water primarily exist as biofilm rather than as planktonic cells (Costerton et al., 1995). Biofilms are formed by microbial communities attaching to the surface of living or inanimate objects (Costerton et al., 1995). All the objects in water, including rocks, plants, and animals, have surfaces that may be occupied by biofilms (Battin et al., 2003). The characteristics of the biofilms are based on the different properties of the objects attached. For instance, most biofilms attached to the rock surface are autotrophic, and the microbial community is primarily composed of algae. Most biofilms formed on decayed animal and plant carcasses are heterotrophic, and the microbial community is primarily composed of bacteria and fungi (Tank and Dodds, 2003; Sinsabaugh et al., 2010). Some researchers have investigated the succession process of the bacterial community in biofilm on submerged animal carcasses compared to microbial communities attaching to ceramic tiles (Dickson et al., 2011; Benbow et al., 2015; He et al., 2019). Studies have shown that the bacterial community structure of the biofilm on the surface of the carcass in water differs in various seasons. The bacterial composition in the biofilm on the carcass also differs significantly from the tiles that sink into the water, displaying a certain succession pattern. However, Dickson et al. (2011) and Lang et al. (2016) took samples every 3–4 days, while Benbow et al. (2015) took samples every week. The relatively longer sampling interval may have missed some important information in the succession pattern of microorganisms. Although He et al. (2019) took samples once a day, the whole experiment lasted only 7 days. The patterns in change of microorganisms thereafter are unknown, which merits further study.

Algae are widely distributed almost everywhere with low environmental conditions and strong adaptability (Casamatta and Verb, 2000). In forensic practice, the diatom test is commonly used to determine whether death occurred due to drowning and can also detect whether the decedent entered the water before or after death (Piette and Letter, 2006). In recent years, researchers have studied the value of algae in estimating the postmortem submersion interval (PMSI). Casamatta and Verb (2000) placed 24 rat carcasses in turbulent streams and gentle rivers and observed species of algae on the carcasses during the decomposition process. They found that the diversity of algae on the carcasses of the two groups increased, with the species of diatoms being the most diverse. Haefner et al. (2004) also observed the growth rates of algae on pig carcasses and tiles by comparing their chlorophyll-a concentration. They found that the growth rates of the former were significantly faster than that of the latter, and the growth rates positively correlated with the sampling time. Zimmerman and Wallace (2010) also observed the diversity of algae on pig carcasses and tile placed in seawater ponds. They found that the abundance of algae negatively correlated with the three decomposition stages.

In recent years, microbial-related research widely uses high-throughput next-generation sequencing (NGS) technologies, such as MiSeq (Illumina) and 454 (Roche). However, the NGS platform generates relatively short sequences, which limits the quality of data. The more recent third-generation sequencing technology improves the accuracy of analyzing microbial communities. The third-generation technology generates long reads, over 10,000 bp using the single-molecule real-time (SMRT) method, with a < 1% average error rate post-annealing with the SMRTbell adapter (Wu et al., 2021). So far, no study has utilized third-generation sequencing technology to study microorganisms associated with decomposition.

This study aimed to evaluate the potential value of insects, RNA, bacteria, and algae for postmortem submersion interval estimation using a rat model. The indicators measured included (1) the succession and development of sarcosaprophagous insects; (2) gene expression from RNA isolated from rat brain tissue; (3) bacterial biofilm communities attached to carcasses, and (4) algal communities from the aquatic habitat where the rat carcasses were submerged.

## Materials and Methods

### Experimental Site

This study was conducted in a small river near the Forensic Autopsy Center of Suzhou, China. Suzhou is located in east China (31°21′ N, 120°53′ E). The altitude is 3–5 m, and it has a subtropical oceanic monsoon climate with an annual mean temperature 16.3°C and a mean annual precipitation of approximately 1,000 mm. The river is primarily formed by rainfall that accumulates in the summer and is oriented south and north. The river flows very slowly, and its movement is barely visible. The bank of the river with a length of 220 m was selected as the experimental area. The width of the river in this area is 4–5 m, and the depth is approximately 1 m. The vegetation in east and west banks of the river are both *Platycladus orientalis* (L.) Franco. The sun will be shaded by the trees in the morning and afternoon and forms a shaded environment in the river. The experiment was conducted in the dry season, and the total rainfall was less than 50 mm during the experiment.

### Experimental Animals

This study used 45 6-week-old specific pathogen-free Sprague-Dawley male rats (*Rattus norvegicus domestica* L.) (Changzhou Cavens Biotechnology Co., Changzhou, China) that weighed approximately 200 g. The rats were killed *via* cervical dislocation at the experimental field site at approximately 10 a.m. on September 29, 2020. The site where the rats were killed was only meters from the river. Each rat was placed in a 30 cm high and 20 cm wide plastic net bag with a mesh size of approximately 8 × 8 mm^2^, and then placed in the river at the experimental site. The mesh of the net bag is elastic. It could adhere to the surface of carcass after they are placed in the bag. The inter-carcass distance was not less than 4 m. The carcasses were placed approximately 1 m from the riverbank and secured with ropes to prevent them from floating away. The animal research followed the regulations of Suzhou University and was approved by the Animal Protection and Use Committee (ECSU-20190000109).

### Temperature Measurement

The air temperature was measured hourly using a Testo l75-H1 thermohygrometer recorder (Germany). The device was equipped 1.2 m above the ground at the field site, avoiding direct sun and sheltered from the rain. A Yowexa YPL-10 waterproof thermometer (China) was first supported by a bracket that stood on the riverbank, and its temperature probe was inserted into approximately 10 cm of water to record the water temperature every 1 h.

### Observation of the Body Decomposition

The degree of carcass decay was observed, recorded, and photographed. The five stages of carcass decomposition that had previously been defined were delimited: submerged fresh stage, early floating stage, floating decay stage, advanced floating decay stage, and sunken remains stage (Zimmerman and Wallace, 2010).

### Observation of Insects on the Carcass

This study selected terrestrial insects rather than aquatic insects as the research object because aquatic insects do not oviposit on the carcasses and thus have little significance to the estimation of PMSI. After the carcass floated, the activities of insects were monitored once a day until day 10 and every other day thereafter until day 18. Fly adults were collected before the rat was picked from the water. The insects were collected using a 25 cm diameter insect net with a 2 m pole, and five back-and-forth sweeping motions were applied to each carcass. After the brain tissue and bacterial biofilm sampling, the immature insects, including egg masses and larvae, were observed and collected with tweezers. Only three carcasses picked from the water were used to investigate the immature insects each time. The carcasses in the river were not disturbed to avoid the effects on the microbial and RNA results. The whole sampling procedure of the insects took no less than 30 min. The collected adult samples were killed using ethyl acetate in bottles, and the larval samples were killed in boiling water ≥ 90°C and then stored in 80% alcohol. The species of the collected insect samples were identified under a microscope using the identification key by Fan (1992).

### Analysis of RNA Degradation in Brain Tissue

Three carcasses were picked from the river each time (*n* = 3). The head skin and skull of the rats were carefully separated using scissors and hemostatic forceps. After the brain tissue was exposed, all the samples were placed in sterile tubes and stored in liquid nitrogen. The samples were taken at day 0 (0 h), day 1 (24 h), day 2 (48 h), day 3 (72 h), day 4 (96 h), day 5 (120 h), day 6 (144 h), day 7 (168 h), day 8 (192 h), day 9 (216 h), day 10 (240 h), day 12 (288 h), day 14 (336 h), day 16 (384 h), and day 18 (432 h). The microbial samples on the skin were obtained immediately after sampling the brain tissues (as described in the following subsection). The rat carcasses were disposed of after the sampling.

A UNIQ-10 column TRIzol total RNA extraction kit was used to extract total RNA from the samples. Agarose gel electrophoresis was used to detect the quality of RNA. A micro spectrophotometer (NanoDrop 2000) was used to determine the concentration of RNA. Purified RNA was treated using DNase I (TaKaRa, Japan). Purified RNA (1 μg) was used for reverse transcription of the first strand of cDNA *via* the PrimeScript™ RT reagent kit (TaKaRa).

Primer Premier 5.0 software was used to design three pairs of real-time fluorescence quantitative PCR primers (Table 1). GADPH, β-actin and 18S were used as the target genes, while 5S was used as the reference gene. The cDNA sample was diluted 10 times using real-time quantitative PCR. A LightCycler480 II fluorescence quantitative PCR instrument (Roche, Rotkreuz, Switzerland) was used to detect the diluted cDNA sample with 2x SG fast qPCR Master Mix reagent (B639271, BBI, Roche). The reaction system had a volume of 20 μl with the following reaction conditions: 95°C for 3 min, 95°C for 5 s, and 60°C for 30 s.

**TABLE 1:** Primer sequences used in RNA and microbial experiments.

	Gene	Forward primer (5′–3′)	Reverse primer (5′–3′)
RNA study	5S	CTACAGCCATACCACCCGG	CGGTATCCCAGGTGGTCTC
	GAPDH	CAAGTTCAACGGCACAGTCAA	CGCCAGTAGACTCCACGACA
	β-actin	CGTAAAGACCTCTATGCCAACA	AGCCACCAATCCACACAGAG
	18S	TTAGTTGGTGGAGCGATTTGT	GGACATCTAAGGGCATCACAG
Microbial study	Full length 16S	AGRGTTTGATYNTGGCTCAG	TASGGHTACCTTGTTASGACTT
	Full length 18S	AACCTGGTTGATCCTGCCAGT	GATCCTTCTGCAGGTTCACCTAC

The ΔCt mean value was calculated as follows: ΔCt = ΔCt _(target gene)_ − ΔCt _(reference gene)_. Origin Pro 8.6 software (OriginLab, Northampton, MA, United States) was used to assess the changes in gene expression with time.

### The Succession Pattern of Microbial Community

Microbial sampling was adopted to the same sampling interval as the RNA study. Water samples (1,000 ml) at a depth of 10 cm from the top within 10 cm of the rat carcass were collected by a Wanzhong WZ-1L hydrophore (China) before the carcasses were removed from the water. The pH of the water was measured with a pH tester (PHTESTER, accuracy of 0.01). The water sample was filtered using filter paper for subsequent analysis of the algae. A large skin area over the whole body was rubbed gently for 60 s using a sterile cotton swab (13 × 100 mm), and a bacterial film sample was obtained on the skin surface. The hair was not removed or parted to reach the skin, since the biofilm communities usually formed between the skin and hair. The sampling procedure was standardized across all the carcasses. After sampling, the water sample and bacterial film were immediately placed in a foam box that contained several ice bags and then stored at −80°C for further analysis.

A DNA Extraction Kit (PowerSoil^®^ DNA Isolation kit) was used to extract the total DNA. The specific primers with Barcodes were synthesized according to the primers in Table 1 and amplified using 96 well PCR (AB: 9902). The reaction system contained 40–60 ng genomic DNA, 2.5 μl of forward and reverse primer, 1 μl KOD FX Neo (TOYOBO), 25 μl KOD FX Neo Buf (2X), and 10 μl dNTP. Distilled water was added to bring the column up to 50 μl. PCR was performed on the target region. The reaction conditions of the bacterial samples were as follows: 95°C for 5 min, 30 cycles at 95°C for 30 s, 50°C for 30 s, 72°C for 1 min, then 72°C for 7 min, and 4°C: ∞. The reaction conditions of the algae samples were as follows: 95°C for 5 min, 35 cycles at 95°C for 30 s, 55°C for 30 s, 72°C for 120 s, then 72°C for 7 min, and 4°C: ∞. ImageJ software (NIH, Bethesda, MD, United States) was used to quantify the amplification products from electrophoresis. After quantification, the samples were mixed (mass ratio, 1:1), then recovered and purified using 0.8X magnetic beads (MagicPure size selection DNA Beads) to form a sequencing library. The library was inspected to ensure quality. The qualified library was sequenced using PacBio Sequel. The CCS (circular consensus sequencing) sequence was exported from the original data. Barcode identification (LIMA version 1.7.0), quality filtration (Trimomatic20, version 0.33), and chimeric sequence identification and removal (Uchime21, version 8.1) were performed to CCS to obtain the optimized CCS sequence.

The sequences were clustered at a similarity level of 97% (USEARCH22, version 10.0). Moreover, 0.005% of the sequences were used as the threshold to filter OTU. R (R Core Team, 2013), and Python (Python Software Foundation, United States) platforms were used to plot the relative abundance histogram, principal coordinate PcoA analysis chart, and the PERMANOVA analysis chart.

## Results

### Environmental Conditions During the Experiment

**FIGURE 1:**
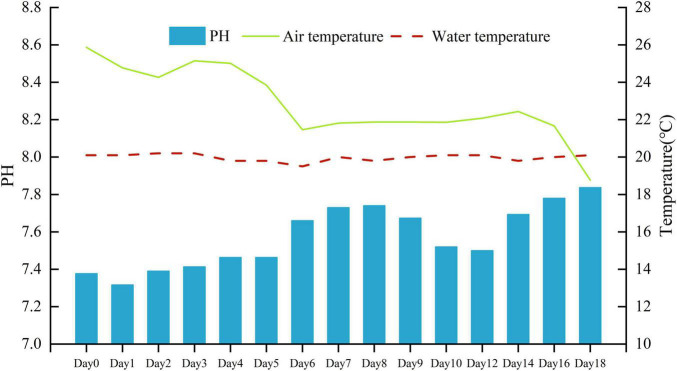
Daily air temperature, water temperature (°C), and water pH during the experiment.

The mean air temperature during the experiment was 22.8°C (lowest, 18.8°C and highest, 25.9°C). The mean water temperature during the experiment was 20.0°C (lowest, 19.5°C and highest, 20.2°C). The pH value in the water gradually increased from 7.32 to 7.84 (Figure 1).

### Decomposition Process of the Carcasses

The carcasses sank into the water immediately after placement and the submerged fresh stage began. Half of the carcasses floated and entered the early floating stage on day 2, and all the carcasses floated on day 3 with bloated bodies. The floating decay stage began on day 6. Minor decay of the carcass was visible, and the loss of muscle mass could be observed on some of the carcasses. The carcass skins were covered with a thin and sticky microbial biofilm during this stage. Major decay of the carcass was observed on day 14. The leg bones and skull of most of the carcasses were exposed. The carcasses entered the advanced decay stage. The carcasses were found still floating during the last sampling on day 18, with approximately 1/3 of the soft tissues missing, and no carcasses entered the stage of sunken remains in this study.

### Observation of Insect Species on the Carcass

Although half of the carcasses floated on day 2, adult flies did not arrive at the carcasses until day 3. The floating carcasses attracted six sarcosaprophagous species, including *Chrysomya megacephala* (Fabricius), *Chrysomya rufifacies* (Macquart), *Lucilia sericata* (Meigen), *Boettcherisca peregrina* (Robineau-Desvoidy), *Musca domestica* Linnaeus, and *Hydrotaea spinigera* Stein. The first insects that arrived at the carcasses were *C. megacephala* and *L. sericata*. They were the most abundant species on the carcasses with a relative abundance of 35.2 and 30.6%, respectively. Both species arrived at the carcasses on day 3, dominated on day 5–day 7, and only a few individuals could be observed after this point. *Chrysomya rufifacies* adults were first collected on day 4 and dominated on days 6–7. They disappeared from the carcasses afterward. Only a small number of *B. peregrina*, *M. domestica*, and *H. spinigera* were observed on the carcasses, with relative abundances of 5.6, 7.4, and 4.6%, respectively. Their first appearance on the carcasses occurred at 7, 7, and 9 days, respectively. Carrion insects only colonized six carcasses. The first oviposition was found on day 5, and egg masses were then found on days 6 and 7. The larvae were found on three carcasses collected on days 8, 10, and 14, and the larvae were identified as *C. megacephala*, *C. rufifacies*, and *L. sericata*. The puparia were not found during the study. The adults of *L. sericata* and *B. peregrina* were collected during the last sampling on day 18, but no immature insects were found on the carcasses. These results showed that insects only appear in large numbers from days 5 to 7 and did not have a regular succession pattern.

### RNA Changes in Brain Tissues With Postmortem Interval

**FIGURE 2:**
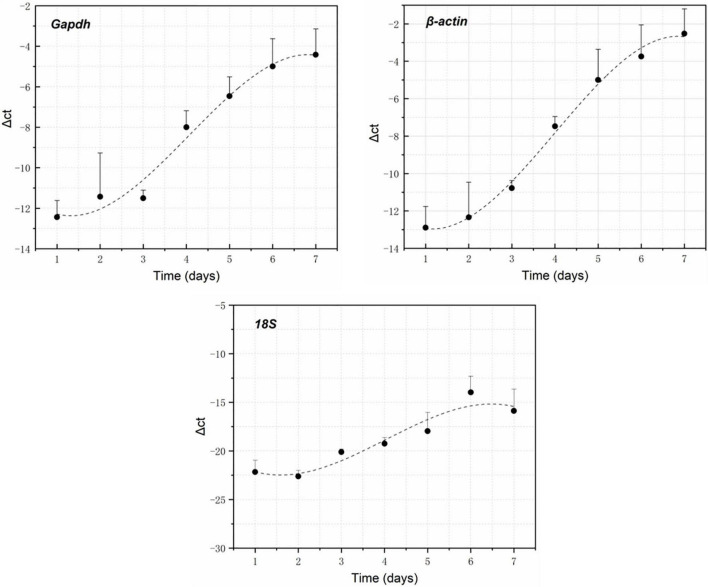
GAPDH, β-actin, and 18S expression changes within 7 days after death using 5S as the reference gene.

RNA electrophoresis showed that the total RNA of brain tissue of the rat carcasses gradually degraded with the extension of PMI from days 0 to 6. The total RNA was severely degraded and decomposed into small molecular fragments after 7 days. Therefore, the relative expression of GAPDH, β-actin, and 18S was detected within the first 7 days (from days 0 to 6). Quantitative PCR showed that all the genes had a regular change trend within 7 days after death. The GAPDH gene increased in gene expression relative to 5S gene, except that its expression was slightly down-regulated on day 2. The gene expression of β-actin positively correlated with the decomposition time. Its relative expression was up-regulated at days 0–6. The relative expression of the 18S gene decreased slightly on day 1, gradually increased from days 2 to 5, and decreased again on day 6, showing an S-shaped curve (Figure 2). A regression analysis was used to establish the corresponding relationship between gene expression and time, with time as the independent variable and ΔCt as the dependent variable. The results showed that the univariate cubic regression equation could more effectively simulate the relationship between gene expression and decomposition time. The correlation coefficient *R*^2^ of each regression model was ≥ 0.924 (Table 2).

**TABLE 2:** Simulation equation of the expression of each gene with decomposition time.

Gene	Equation	*R* ^2^
GAPDH	GE = −0.096t^3^ + 1.1718t^2^ − 2.5933t − 10.769	0.977
β-actin	GE = −0.1117t^3^ + 1.3422t^2^ − 2.661t − 11.507	0.995
18S	GE = −0.1249t^3^ + 1.5107t^2^ − 3.8512t − 19.674	0.924

*GE and t indicate the relative expression and decomposition time.*

### Change Pattern of Bacterial Biofilm Communities on the Carcass Skin Surfaces

A total of 42 skin swab samples were collected from the carcass surface (Day 9 samples produced substandard results as the samples of skin on day 9 were unqualified). A total of 516,684 CCS sequences were obtained after barcode identification. Each sample produced at least 9,292 CCS sequences, with an average of 11,482 CCS sequences. A total of 463,338 effective CCS sequences were obtained after length filtration and chimera removal. The CSS sequences were divided into 740 OTUs after cluster analysis.

**FIGURE 3:**
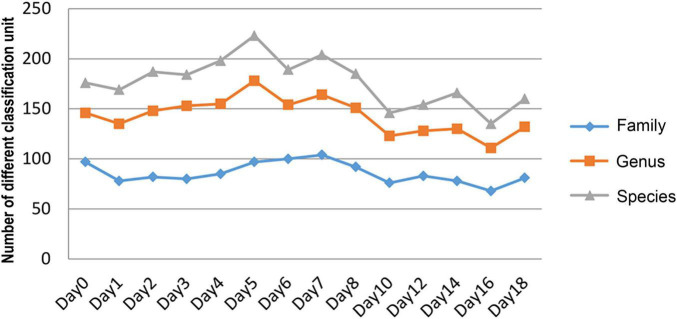
The number of changes of different classification units (family, genus, and species) with time during decomposition.

**FIGURE 4:**
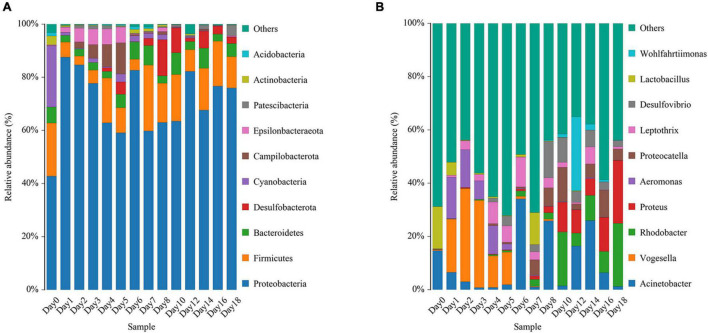
Variation in the bacterial community structure on the skin surface during decomposition at the phylum level **(A)** and genus level **(B)** (only the top 10 most abundant).

The relative abundance changes in bacteria were detected at the phylum level, family level, and genus level to determine the changes in bacterial community structure on the rat skin biofilm during decomposition. The sequencing results showed that the bacteria had 24 phyla, 186 families, 375 genera, and 510 species. Bacterial diversity showed a downward trend from days 0 to 1, increased from days 1 to 5 and gradually decreased after day 5 (Figure 3). Proteobacteria, Firmicutes, and Bacteroidetes existed during the whole decomposition process, while Fusobacteriota and Chloroflexi only appeared during the first 7 days at the phylum level. The abundance of Proteobacteria first decreased and then gradually increased. Desulfobacterota, Campilobacterota, Patescibacteria, Bdellovibrionota, Deinococcus-Thermus, Nitrospirae, and Fusobacteria appeared from the second day of sampling. Fusobacteria only appeared on days 2–5 (Figure 4A). *Vogesella* was more abundant on days 1–5, and *Aeromonas* was more abundant on days 1–4 at the genus level. Moreover, *Proteus* and *Proteocatella* increased in abundance after day 7 (Figure 4B).

**FIGURE 5:**
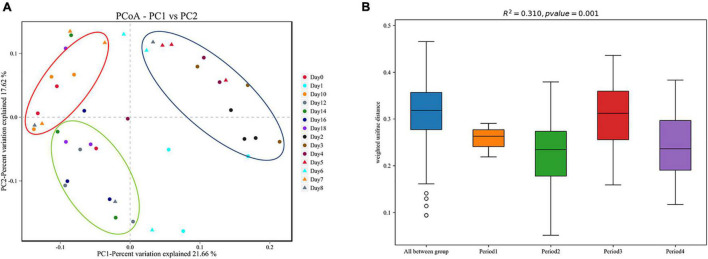
Beta diversity analysis of skin swab samples. **(A)** Pcoa-PC1 vs. PC2, **(B)** PERMANOVA analysis results. Period 1, Period 2, Period 3, and Period 4 indicate day 0, days 1–6, days 7–10, and days 12–18, respectively.

Beta diversity analysis showed that the bacterial composition in the skin biofilm was associated with the decomposition time (Figure 5A). PCoA analysis based on weighted UniFrac showed that the bacterial composition from days 0 to 18 could be clustered into four periods (period 1 to period 4). The bacterial composition of day 0 was different from those of the other sampling times. The bacterial communities from days 1 to 6, days 7 to 10, and days 12 to 18 could be separated, since three distinct clusters were formed. A PERMANOVA analysis was performed on the analytical results of PCoA to test whether the differences among samples in the four groups were significant. The analysis supported the clustering and confirmed that the bacterial communities of each period were different (*R*^2^ = 0.310, *P* = 0.01) (Figure 5B).

### Change Pattern of Algal Communities From the Aquatic Habitat Where the Rat Carcasses Were Submerged

There were 27 filtered water samples (the samples after day 9 were missing during the delivery for DNA sequencing). A total of 399,269 CCS sequences were obtained after Barcode identification. Each sample produced at least 6,702 CCS sequences, with an average of 14,788 CCS sequences. A total of 336,125 effective CCS sequences were obtained after length filtration and chimera removal. The CSS sequences were divided into 581 OTUs after cluster analysis.

**FIGURE 6:**
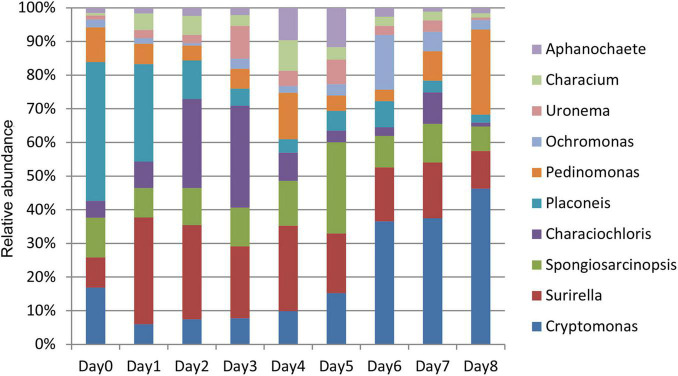
Histogram of relative abundance of algae in the water around carcass at the genus level.

This study focused on the relative abundance changes of algae at the genus level to determine the changes in algae in the water around the carcass during the decomposition process. The relative abundance changes of 10 algae were compared with top abundance at the genus level from days 0 to 8 (Figure 6). The relative abundance of each genus had a certain change pattern during the decomposition process, providing a basis for estimating the decomposition time of carcass in the water.

**FIGURE 7:**
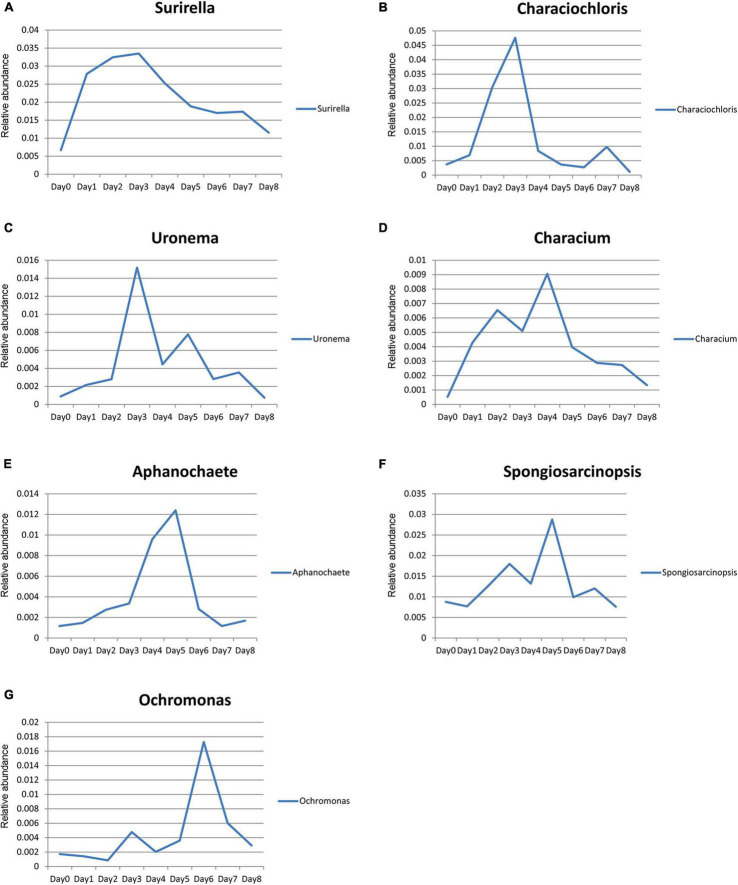
Relative abundance changes of seven species of algae with time. **(A)** *Surirella*; **(B)** *Characiochloris*; **(C)** *Uronema*; **(D)** *Characium*; **(E)** *Aphanochaete*; **(F)** *Spongiosarcinopsis*; **(G)** *Ochromonas*.

The relative abundance of *Surirella*, *Characiochloris*, *Uronema*, *Characium*, *Aphanochaete*, *Ochromonas*, and *Spongiosarcinopsis* first increased and then decreased with time (Figure 7). In particular, the relative abundance of *Surirella*, *Characiochloris*, and *Uronema* gradually increased from days 0 to 3 and then gradually decreased. The abundance of *Characium* peaked on day 4. The *Aphanochaete* and *Ochromonas* abundances peaked on day 5. *Aphanochaete* had a good asymptotic trend of increasing and decreasing, while *Ochromonas* first increased, then decreased. The abundance of *Spongiosarcinopsis* increased on days 0–6 and then began to decrease.

**FIGURE 8:**
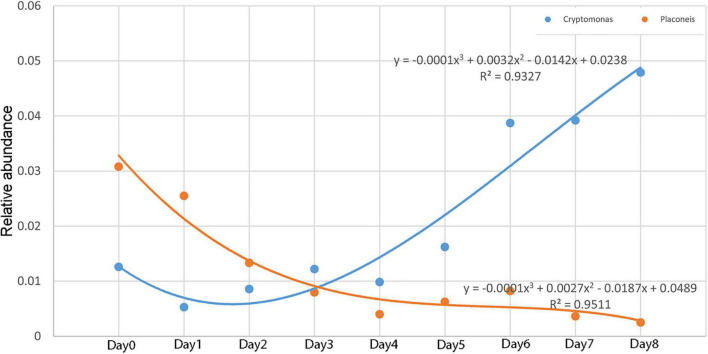
Relative abundance changes of *Cryptomonas* and *Placoneis* with time.

The relative abundance of *Cryptomonas* and *Placoneis* showed a monotonous trend with time. The relative abundance of *Cryptomonas* increased with time, while that of *Placoneis* decreased. The change in the relationship between relative abundance and decomposition time (Figure 8) was plotted using the relative abundance values of *Cryptomonas* and *Placoneis* as dependent variables and different sampling times as independent variables. A regression analysis was then conducted to obtain the simulation equation. The regression coefficient *R*^2^ of the equation was >0.9, indicating that the data fit well.

## Discussion

This study showed that the carcass decomposed slowly in water. Approximately 2/3 of the soft tissues still remained on the last sampling on day 18. There are two reasons that may explain this. First, most parts of the carcass tissues were submerged in water, and flies could only colonize a small part of the body, thus, fewer fly eggs were oviposited (Heo et al., 2008). Fly larvae are one of the primary organisms that facilitate decomposition in water (Dalal et al., 2020). A reduced number of maggots greatly decrease the decomposition of carcasses. Secondly, the small mesh size of the net bag (8 mm) only let small-sized shrimp and fish access the carcass, and aquatic organisms that are larger in size were largely excluded, which slowed the decomposition rate.

GAPDH, β-actin, and 18S RNA are housekeeping genes that are widely distributed in organisms and are highly conserved and stable (Thellin et al., 1999). This study used a univariate cubic regression model to simulate the changes in expression of GAPDH, β-actin, and 18S gene in the rat brain tissues from days 0 to 6 after death. The expression changed with time, consistent with the findings of Liu et al. (2007); Ren et al. (2009), Zhu et al. (2011); Elghamry et al. (2017), and Lv et al. (2017). The RNA collected from the carcass in water varied in a similar pattern to that on land. In addition, the RNA degradation of water carcass might have higher and more reliable repeatability of the results than that of the land carcass. Water has a higher specific heat capacity, and the water temperature during the experiment is relatively constant (19.5–20.2°C) and less affected by the ambient temperature. This could also be related to the relatively shady water environment, which is less affected by solar radiation. Previous studies have shown that the rate of degradation of RNA is slower at low temperatures and faster at high temperatures (Zhu et al., 2011; Lv et al., 2016a,b, 2017). The reliability of the PMI results could be better when the RNA degradation data obtained under constant temperature conditions are used in the water environment with a relatively constant temperature.

Herein, third-generation full-length sequencing technology was used for the first time to confirm whether there was a certain succession pattern of bacteria on the skin surface of the carcass in water and algae in water samples around the carcass. This could provide a basis to estimate the PMI of carcass in water. Bacterial diversity showed a downward trend from days 0 to 1, possibly owing to the drastic changes in environment. Before the rat was sacrificed, the bacteria on the skin surface of the rats were primarily those adapted to the aerobic environment. After the carcass was placed into the river, those primary aerobic bacteria gradually died in the anaerobic environment. The aerobic bacteria in water had not yet fully developed and attached to the surface of the carcass, causing the decrease in bacterial diversity. After day 1, the bacterial diversity gradually increased, and some new bacterial communities, probably the bacteria from water, were detected throughout the analysis.

Proteobacteria, Firmicutes, and Bacteroidetes were identified during the decomposition of carcass, consistent with other relevant studies (Dickson et al., 2011; Benbow et al., 2015; He et al., 2019). Moreover, Proteobacteria had the highest relative abundance. Benbow et al. (2015) and He et al. (2019) showed that the relative abundance of Proteobacteria gradually decreases, while that of Firmicutes gradually increases during the decomposition process, inconsistent with this study. The inconsistency could be because the study of Benbow et al. (2015) lasted for 28 days, and the samples were collected once a week. The change in intermediate abundance may not be observed when the sampling intervals were too long. Although He et al. (2019) took samples every day, the experiment lasted for only 7 days. Some specific bacteria with low abundance also showed a certain succession pattern, which can also indicate PMI. Fusobacteriota and Chloroflexi only appeared during the first 7 days, consistent with the findings of Dickson et al. (2011) and thus, can be used to determine whether the PMSI exceeds 1 week. Fusobacteria only appeared on days 2–5, and can describe the narrower PMSI. The simultaneous detection of Tenericutes and Bdellovibrionota can detect the PMSI on day 6 *via* the comprehensive application of different bacteria. Chloroflex and Fusobacteria were detected simultaneously. Combined with the carcass decomposition phenomena, the PMSI could be locked on days 2 and 5.

Herein, a few algae species were detected compared with other algal experiments (Casamatta and Verb, 2000; Haefner et al., 2004; Dickson et al., 2011). This could be because the small river selected for the experimental site was formed by rainfall and summer ponding. In addition, samples after day 9 were missing, and thus, the algae species after that were unclear. Casamatta and Verb (2000) detected only five species of algae on the 3rd and 8th day of carcass decomposition. However, they identified more algae species on the 12th day of carcass decomposition.

The existing research on the relationship between algae and PMSI is based on the algae on the carcass biofilm (Casamatta and Verb, 2000; Haefner et al., 2004; Dickson et al., 2011). Early experimental studies found that salmon carcasses in freshwater can release a large amount of nitrogen, thus, accelerating the growth rate of algae in the surrounding waters (Richey et al., 1975; Parmenter and Lamarra, 1991). Inspired by these results, we hypothesized that the algae in the water around the carcass may have regular succession patterns that can be used to estimate the PMSI. Thus, the preliminary design of this experiment included algae as a potential indicator. Unlike previous studies that identified the algae using the morphology method (Casamatta and Verb, 2000; Zimmerman and Wallace, 2010), this study adopted third-generation full-length sequencing technology to directly obtain the relative abundance of algae in each sample using 18S rRNA primers. This study compared the 10 algae that were the most abundant at the genus level. The results showed that the relative abundance of algae did have a specific change pattern, indicating that the process of carcass decomposition impacted the growth rate of algae, thus providing a basis for estimating the PMSI. Furthermore, *Cryptomonas* and *Placoneis* had a valuable monotonic change trend. In particular, the relative abundance of *Placoneis* and the decomposition time had a good polynomial fitting degree, with an *R*^2^-value of 0.95, thus enhancing the accuracy and providing a basis for PMSI.

The study was conducted in an area with rivers of various lengths that crisscross like a spider web. Corpses often occurred in the rivers, and PMSI is always a problem for the investigators. This study selected the river as the aquatic environment, aiming to provide a feasible method to estimate PMSI in a similar environment. However, this study was conducted in a small river, which may differ from a larger river in temperature, flow rate, depth, and composition of aquatic organisms. Whether these variables would have an impact on insects, RNA and microorganisms merits further study. We found that the net bags are lightweight, and carcasses can float above the water naturally, which is suitable for placing the carcasses. However, the accessibility of terrestrial insects and aquatic organisms to the carcasses was affected owing to the small mesh. A net bag with a larger mesh size should be used in the future to allow differently sized aquatic organisms and terrestrial insects to access the carcass and simulate an environment more similar to that of nature. In this study, terrestrial insects were selected as the research objects, since most aquatic organisms do not oviposit on the carcass. It is difficult to establish a connection between the occurrence of aquatic organisms with the PMSI. However, from an ecological perspective, each carcass should be regarded as a micro-ecosystem, and all organisms that occurred in the ecosystem should be fully studied and analyzed, so as to provide a more reliable PMSI. This is a preliminary study that used rat carcasses as the decomposition model. Studies have found that the body size is a factor of key importance for decomposition and carrion entomofauna (Matuszewski et al., 2014, 2016; Wang et al., 2017). A regular succession pattern of insects was not found in this study probably owing to the small body size of the rats. In future study for practical use, human corpses or animal carcasses that are close to human corpses should be selected.

In conclusion, this study revealed that submerged carcasses have a simple insect composition and attracted only six species of flies. The flies did not have a regular succession pattern and colonized only six carcasses, indicating that terrestrial insects might not be suitable for estimating PMSI. The RNA isolated from rat brain tissue positively correlated to the decomposition time. It can delineate the range of PMSI within the first 7 days. The bacteria on the skin surface of the carcass and the algae in the water samples around the carcass have a succession pattern that corresponds to decomposition time, suggesting further application in forensic science in aiding to determine PMSI. This is the first study to assess algae in water samples around a carcass using third-generation sequencing technology. However, the changes in rate of algae growth in the late stage of decomposition should be further analyzed owing to the missing samples.

## Data Availability Statement

The datasets generated for this study have been uploaded to GenBank by the accession numbers RJNA777763 and PRJNA777773.

## Ethics Statement

The animal study was reviewed and approved by the regulations of Suzhou University and was approved by the Animal Protection and Use Committee (ECSU-20190000109).

## Author Contributions

YW and MW: investigation, methodology, funding acquisition, and writing – original draft. WX, YZ, and YW: investigation and data analysis. JW: supervision, funding acquisition, and writing review and editing. All authors contributed to the article and approved the submitted version.

## Conflict of Interest

The authors declare that the research was conducted in the absence of any commercial or financial relationships that could be construed as a potential conflict of interest.

## Publisher’s Note

All claims expressed in this article are solely those of the authors and do not necessarily represent those of their affiliated organizations, or those of the publisher, the editors and the reviewers. Any product that may be evaluated in this article, or claim that may be made by its manufacturer, is not guaranteed or endorsed by the publisher.
